# Factores asociados y tipos de lesiones oculares ocurridas en la práctica odontológica: revisión sistemática

**DOI:** 10.21142/2523-2754-1103-2023-169

**Published:** 2023-09-26

**Authors:** Abanto Sheron Jordi, Martín Andrés Chávez Méndez

**Affiliations:** 1 Departamento de Ciencias de la Salud, Carrera de Estomatología, Universidad Científica del Sur. Lima, Perú. 100028618@cientifica.edu.pe Universidad Científica del Sur Departamento de Ciencias de la Salud Carrera de Estomatología Universidad Científica del Sur Lima Peru 100028618@cientifica.edu.pe; 2 Departamento de Rehabilitación Oral, Carrera de Estomatología, Universidad Científica del Sur. Lima Perú. mchavezme@cientifica.edu.pe Universidad Científica del Sur Departamento de Rehabilitación Oral Carrera de Estomatología Universidad Científica del Sur Lima Peru mchavezme@cientifica.edu.pe

**Keywords:** lesiones oculares, odontólogo, práctica odontológica, accidentes oculares laborales, eye injuries, dentist, dental practice, occupational eye accident

## Abstract

**Introducción:**

El odontólogo y personal auxiliar están en constante riesgo de enfermedades ocupacionales, como alergias, infecciones, lesiones cutáneas u oculares que podrían afectar a los pacientes. Un problema de interés social son las lesiones oculares que le ocurren al personal odontológico. La literatura revela que estas lesiones están asociadas a partículas sólidas y salpicaduras de fluidos. Nuestro objetivo fue, mediante una revisión sistemática, identificar los tipos de lesiones oculares y sus factores asociados ocurridos en la práctica odontológica.

**Materiales y métodos:**

Los estudios relevantes se buscaron en las bases de datos PubMed, Scopus, Ebsco, entre otras. La búsqueda incluyó estudios transversales que evaluaban la evidencia sobre lesiones oculares ocurridas en la práctica odontológica, y se excluyeron las revisiones de literatura, cartas al lector y editoriales.

**Resultados:**

Se incluyeron 8 estudios para desarrollar la revisión sistemática y se determinó que los factores asociados con las lesiones oculares ocurridas en la práctica odontológica son el uso ocasional de protección ocular, la edad, el sexo y los años de práctica, mientras que los tipos de lesiones oculares son la entrada de líquidos y de cuerpos extraños.

**Conclusiones:**

La deficiencia en el cumplimiento de protocolos de protección ocular o facial en la práctica dental origina las lesiones oculares, por la entrada de líquidos, de cuerpos extraños, salpicaduras de fluidos biológicos o conjuntivitis. Estas lesiones oculares se generan a partir de factores asociados como el uso ocasional de protección ocular, la edad, el sexo, entre otros.

## Abstract (en)

**Introduction:**

The dentist and auxiliary personnel are in constant risk of occupational diseases such as: allergies, infections, skin or eye injuries; that could affect patients. A problem of social interest are the ocular injuries that occur to dental personnel. The literature reveals the prevalence of eye injuries in dental practice that are associated with solid particles, fluid splashes. Our objective was, through a systematic review, to review the types of ocular injuries and their associated factors that occurred in dental practice.

**Material and Methods:**

The relevant studies were searched in the databases: PubMed, Scopus, Ebsco, among others. The search included cross-sectional studies that evaluated the evidence on ocular injuries occurring in dental practice, excluding literature reviews, letters to the reader, and editorials.

**Results:**

Eight studies were included to develop the systematic review; determining that the associated factors of ocular injuries occurred in dental practice are: a) occasional use of ocular protection b) age c) sex d) years of practice and the types of ocular injuries are the entry of liquids and foreign bodies.

**Conclusions:**

Deficiency in compliance with eye or facial protection protocols in dental practice, cause eye injuries; Type of fluid entry, foreign bodies, splash of biological fluids or conjunctivitis. These eye injuries are generated from associated factors such as the occasional use of eye protection, age, sex, and others.

## INTRODUCCIÓN

La odontología es una ocupación que requiere una gran exigencia física y mental, debido a los cambios y competencias en la vida moderna. Con respecto a las exigencias físicas, estas incluyen buena agudeza visual, auditiva, destreza manual, entre otras. Los odontólogos requieren cualidades como resolución de problemas, liderazgo, comunicación, destreza y habilidades de gestión. Al producirse una falta de estas cualidades, podría afectar su desempeño ^1^.

Por otro lado, el personal dental se expone en el ambiente donde labora a microorganismos patógenos que incluyen bacterias y virus que se encuentran en la cavidad oral y el tracto respiratorio. Estos patógenos podrían transmitirse por contacto directo e indirecto ^2^. Incluso, el odontólogo y el personal auxiliar en la actividad profesional están en constante riesgo de enfermedades ocupacionales como alergias, infecciones sistémicas, toxicidad, trastornos músculo esqueléticos, lesiones cutáneas u oculares ^1^.

Los traumatismos oculares son la principal etiología en los servicios oftalmológicos de emergencia en todo el mundo ^3^. Estos pueden tener efectos graves a corto y largo plazo; a menudo, los síntomas se relacionan con el tipo y grado de trauma, presencia de dolor, lagrimeo y visión borrosa. Las lesiones suelen ser dolorosas y generan daños a la vista, especialmente cuando hay desprendimiento de la retina o si la córnea y la esclerótica están afectadas ^4^.

En la actualidad, las personas infectadas de SARS-CoV-2 lo transmiten a otros individuos a través secreciones contaminadas que expulsan cuando hablan, tosen o estornudan ^5^. El órgano de la vista representa una fuente de transmisión por medio de secreciones infectadas, además de ser un vehículo de infección a través de partículas en aerosol y gotitas respiratorias, cuando entran en contacto con la conjuntiva ^6^.

Inclusive, el uso de dispositivos móviles y teléfonos inteligentes ha generado un impacto significativo en la vida diaria de las personas, ya que permiten al usuario navegar por la web, ver videos, chatear en las redes sociales y realizar cursos virtuales ^7^. Pero es cada vez mayor tiempo dedicado a ver estas pantallas ha generado problemas de salud, como fatigas oculares, dolor dentro y alrededor de los ojos, visión borrosa y dolor de cabeza ^8^.

Las lesiones oculares del personal odontológico se han convertido en una problemática de interés social. La literatura al respecto revela que la prevalencia de las lesiones oculares en odontología podría estar asociada con partículas dentales sólidas, como tejidos dentales, cálculos, salpicaduras de sangre y gotas de productos químicos ^2^. Además, existe una revisión sistemática de los tipos de lesiones oculares y factores asociados ^9^^)^ en diversas ocupaciones; pero no han sido reportados aquellos factores relacionados con la práctica odontológica. Por tanto, realizar este estudio generará una mejor perspectiva sobre los riesgos de lesiones oculares y su prevención, mediante el uso de objetos de protección ocular por parte del personal odontológico. 

El objetivo de la investigación fue revisar los tipos de lesiones oculares y sus factores asociados ocurridos en la práctica odontológica, a través de una revisión sistemática de tipo bibliográfico.

## MATERIALES Y MÉTODOS 

Esta revisión sistemática evaluó artículos con diseños descriptivos sobre los factores asociados y tipos de lesiones oculares ocurridas en la práctica odontológica. Se empleó la herramienta SPIDER (^10^) (Sample, Phenomenon of Interest, Design, Evaluation, Research type) para la construcción de la estructura de búsqueda. 

Los estudios relevantes se buscaron en las bases de datos PubMed, Scopus, Ebsco, LILACS, SciELO, Wiley Online Library, Google Scholar, Repositorio Nacional Digital Alicia y revistas no indexadas, usando palabras claves como *ocular injuries*, *eye injuries*, *orbit injuries*, *ocular hazard*, *dentistry*, *dentists*, *dental practice*, *occupational injuries*, *occupational ocular accidents*, así como operadores booleanos y términos MeSH. La búsqueda incluyó estudios transversales en idioma inglés, español y portugués, sin restricción de año ni estado de publicación, pero se excluyeron las revisiones de literatura, cartas al lector y editoriales. 

La investigación se ejecutó de acuerdo con la guía PRISMA ^11^ para la obtención la data final. Luego, cada revisor eliminó los duplicados y procedió a la filtración por título y resumen, además de los criterios de inclusión y exclusión. Para obtener los artículos finales, se tuvieron en cuenta los siguientes datos: autor, año, país, muestra, diseño del estudio, factores asociados y tipo de lesión. 

Por otro lado, ambos investigadores hicieron las revisiones y confirmaron que los artículos seleccionados fueron elegidos correctamente, previa calibración con el índice Kappa ^12^.

La evaluación de la calidad de los estudios incluidos fue realizada con la escala de Newcastle-Ottawa (NOS), adaptada para estudios transversales ^13^, por dos revisores; primero individualmente y luego en conjunto. La puntuación de la escala (NOS) se estableció entre 0-9 ^13^. Asimismo, cada revisor hizo una valoración de los estudios incluidos mediante la clasificación Muy bueno: 9-10 puntos; Bueno: 7-8 puntos; Satisfactorio: 5-6 puntos; e Insatisfactorio: 0 a 4 puntos. Toda esta información se obtuvo de los manuales de Cochrane y Newcastle-Otawa ^14^. Además, la calidad metodológica de los estudios cualitativos incluidos fue evaluada de la misma manera que el riesgo de sesgo, individualmente y luego en conjunto. Se empleó la lista de verificación cualitativa Critical Appraisal Skills Programme (CASP) ^(15, 16)^, la cual consta de 10 preguntas (Tabla 1).

**Tabla 1:** Lista de verificación cualitativa Critical Appraisal Skills Programme (CASP)

1. ¿Hubo una declaración clara de los objetivos de la investigación?
2. ¿Es apropiada una metodología cualitativa?
3. ¿El diseño de la investigación fue apropiado para abordar los objetivos de la investigación?
4. ¿La estrategia de reclutamiento fue apropiada para los objetivos de la investigación?
5. ¿Se recopilaron los datos de una manera que abordará el tema de la investigación?
6. ¿Se ha considerado adecuadamente la relación entre el investigador y los participantes?
7. ¿Se han tenido en cuenta las cuestiones éticas?
8. ¿Fue suficientemente riguroso el análisis de datos?
9. ¿Hay una declaración clara de los hallazgos?
10. ¿Qué valor tiene la investigación?

Para esta revisión no se desarrolló ninguna escala de calibración, pero cada ítem evaluado recibió una puntuación: Sí (1 punto), No sé (0,5 puntos) y No (0 puntos). Por ello, cuando se obtuvo un “Sí” en dos tercios de las secciones del CASP, este se calificó como alta; cuando la puntuación estuvo entre cuatro y seis “Sí”, se consideró moderada y, por último, si más de dos tercios de las respuestas eran “No”, se registró como baja ^17^.

## RESULTADOS

### Resultados de la búsqueda

**Figura 1:**
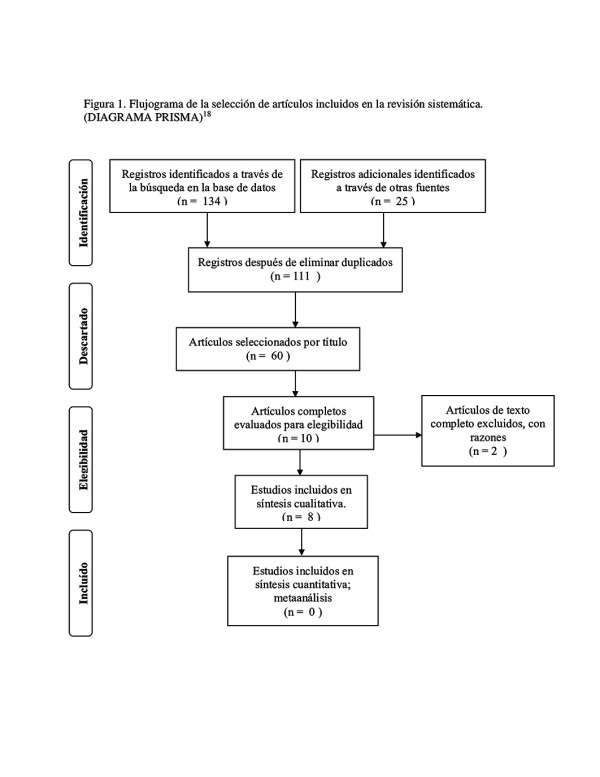
Flujograma de la selección de artículos incluidos en la revisión sistemática (diagrama PRISMA) ^18^

De las bases de datos descritas, se obtuvieron 134 publicaciones y 25 de otras fuentes. De ese total, se excluyeron los duplicados y quedaron 111. Asimismo, se eliminó por título, y quedaron 60 documentos, de los cuales 50 fueron excluidos porque no cumplieron con los criterios de inclusión y exclusión. Quedaron 10 artículos que cumplieron con los criterios de elegibilidad para ser evaluados por texto completo; no obstante, 2 de ellos fueron descartados. Finalmente, 8 estudios fueron los seleccionados y sus datos se registraron electrónicamente en forma detallada (Figura 1).

### Descripción de los estudios incluidos 

Se incluyeron ocho estudios para desarrollar la revisión sistemática, 8 (100%) corresponden a estudios de diseño transversal y presentan los siguientes tipos: 1 retrospectivo (12,5%), 1 multicéntrico (12,5%), 1 de prevalencia (12,5%) y el resto no especifica. Con relación a la base de datos, 6 estudios provienen de la base PubMed, 1 de Scopus y 1 de Hand Searching. Además, se verificó los estudios con la escala de Newcastle-Ottawa, en la cual deberían obtener una valoración tomando en cuenta los diferentes criterios de evaluación (Tabla 2).

Se encuentra la descripción detallada de las características de los estudios seleccionados, por ejemplo, las diferentes bases de datos, el lugar de origen, el año, los autores, el título, el tamaño de la muestra, el diseño de estudio, el periodo de años examinados y el instrumento para la recolección de datos.

**Tabla 2:** Resumen de criterios de Newcastle-Ottawa

Estudio	Selección	Comparabilidad	Exposición	Puntaje	Clasificación	Base de datos
Porter, 1990	1	2	2	5	Satisfactorio	PubMed
Sims, 1993	1	2	3	6	Satisfactorio	PubMed
Zarra, 2012	4	2	3	9	Muy bueno	PubMed
Bhat, 2014	3	1	3	7	Bueno	Hand Searching
Azodo, 2015	4	1	3	8	Bueno	Scopus
Alsabaani, 2017	4	2	3	9	Muy bueno	PubMed
Vanita, 2019	0	1	3	4	Insatisfactorio	PubMed
Aydil, 2020	4	2	3	9	Muy bueno	PubMed

**Tabla 3:** Características de las lesiones oculares

N.º	Referencia, país	Tamaño de la muestra	Periodo de años examinados	Diseño de estudio	Factor(es) asociado(s) con la lesión	Tipos de lesión	Objetivo
1	Porter, RU	N = 302 personal que trabaja en el Bristol Dental Hospital (BDH)	1980-1988	Transversal, retrospectivo	Uso ocasional de protección ocular, exposición a riesgos laborales.	Entrada de líquidos, cuerpos extraños o partículas	Determinar el número y los tipos de accidentes laborales notificados en el Bristol Dental Hospital (BDH) entre los años 1980 y 1988, inclusive.
2	Sims, RU	N =159 consultores de ortodoncia del NHS y 203 especialistas en ortodoncia	Registros de 1990 de cada organización	Transversal	Uso ocasional de protección ocular, exposición a riesgos laborales, fuerzas mecánicas, exposición a riesgo biológico.	Cuerpos extraños, lesión química	Determinar el uso de protección ocular y la incidencia de traumatismos oculares en la práctica de la ortodoncia.
3	Zarra, Grecia	N = 123 endodoncias	2007-2012	Transversal	Uso ocasional de protector ocular, exposición a riesgos laborales, edad, sexo, años de practica	Salpicadura de fluidos biológicos, cuerpos extraños	Investigar entre los endodoncistas griegos la incidencia de accidentes oculares durante la práctica, las circunstancias asociadas a ellos, las medidas terapéuticas tomadas después de los accidentes, su cumplimiento con el uso de protección ocular y su comportamiento de cuidado ocular.
4	Bhat, India	N = 152 personal dental	2012	Transversal	Exposición a riesgo biológicos	Conjuntivitis, gripe ocular	Evaluar el conocimiento y la conciencia sobre la gripe ocular entre los dentistas y auxiliares dentales de la ciudad de Udaipur, Rajastán, India.
5	Azodo, Nigeria	N = 185 cirujanos maxilofaciales	2010-2011	Transversal	Edad, años de práctica, uso ocasional de gafas de seguridad	Cuerpos extraños o partículas Salpicadura de fluidos biológicos	Determinar la prevalencia y el patrón de salpicaduras oculares y cuerpos extraños entre los cirujanos dentales en Nigeria.
6	Alsabaani, Arabia Saudita	N = 233 odontólogos	Años no especificados	Transversal multicéntrico	Ausencia de calificación en posgrado, uso ocasional de protección ocular, horas de trabajo prolongadas	Cuerpos extraños, salpicadura de fluidos biológicos	Evaluar la prevalencia, el patrón y los determinantes de los incidentes oculares y establecer el uso de protección ocular por parte del personal dental en el suroeste de Arabia Saudita.
7	Vanita, India	N =150 odontólogos	Años no especificados	Transversal (prevalencia)	Horas de trabajo prolongadas, posición de practica sentado/parado	Conjuntivitis, abrasión corneal, lesión química	Evaluar la difusión de los TME y los problemas relacionados con la salud ocular entre los cirujanos dentales en la ciudad de Salem, Tamil Nadu, India, según la edad, el sexo y el número de horas de práctica por semana.
8	Aydil, Turquía	N = 26 cirujanos residentes	2018	Transversal	Uso ocasional de protección ocular, educación/no educación en lesiones	Laceraciones, salpicadura de líquidos biológicos, cuerpos extraños	Determinar la prevalencia de lesión e infección ocular entre cirujanos orales y maxilofaciales durante procedimientos ambulatorios.

### Características de los resultados

En la tabla 3, se describen las características de las lesiones oculares incluyendo factores, tipos y objetivo de los estudios seleccionados. Se identificó los factores asociados de las lesiones oculares ocurridas en la práctica odontológica: a) uso ocasional de protección ocular en procedimientos desunión, adhesión, grabado de esmalte, cambios de arco, soldadura, recorte acrílico y otros; b) edad; c) sexo; d) años de práctica; e) horas de trabajo prolongadas; f) ausencia de calificación en posgrado; g) posición de practica sentado/parado; h) exposición a riesgos laborales (rociar agua y saliva en la cara del operador debido a la mala angulación del chorro de agua en la escupidera, desconectar el tubo de succión, limpiar el material de impresión dentales, limpiarse los ojos con los dedos cubiertos de cera o cuerpos extraños, también procedimientos quirúrgicos: abrir paquetes de suturas, infiltración de solución anestésica local, irrigación de los alvéolos infectados, biopsia, irrigación de examen oral, sutura, alisado/legrado radicular, pacientes en posición decúbito supino, uso de compuestos fotopolimerizables, recortes de retenedores termoplásticos, ajuste los arcos de tracción extraorales, error de refracción en la cirugía, fractura de instrumentos de mano, manipulación de agentes esterilizantes químicos para limpieza de equipos). Incluso, en procedimientos endodónticos (uso de agujas de irrigación Luer Lock, uso de lentes de aumento) y procedimientos restaurativos (extracción de amalgama, recorte de prótesis, corte de alambre interdental, pulir dientes); i) exposición a riesgos bilógicos: VIH, COVID-19, contacto directo con personas infectadas, entorno antihigiénico, fatiga visual, virus, bacterias, alérgenos e irritantes, factores ambientales (fumar, polvo, polen); j) fuerzas mecánicas: fractura de cartucho de anestesia. Con respecto al uso ocasional de protección ocular, los cirujanos maxilofaciales utilizaron en procedimientos como: raspado, preparación del diente/cavidad, pulido y extracción del diente con un fórceps.

Se identificó que los tipos de lesiones oculares fueron por:

- a.Entrada de líquidos; clorhexidina, alcohol, solución anestésica local, enjuague bucal de benzalconio, solución salina, suero, agua, liquido modelador, detergente
- b. Cuerpos extraños o partículas: fragmentos dentales, restauraciones, adhesivo, polvo de acrílico, cemento de banda, alambre de ligaduras, amalgamas, NaOCl, EDTA, material provisional, vidrio, fragmento de instrumento, cálculos, óxido de zinc, eugenol, pasta de pulir, metal, arenilla de conducto roto, contacto con guantes
- c. Lesión química: solución de grabado, agente esterilizador
- d. Salpicadura de fluidos biológicos: saliva, sangre o mixto, líquido quístico
- e. Conjuntivitis/gripe ocular
- f. Laceraciones

### Evaluación de la calidad metodológica de los estudios incluidos

Para evaluar la calidad metodológica de los artículos incluidos, se empleó la lista de verificación cualitativa Critical Appraisal Skills Programme (CASP) ^(15, 16)^, que evalúa la validez, los resultados y su aporte final. La valoración de los estudios fue realizada por dos revisores, primero individualmente y luego en conjunto.

Seis artículos exhibieron calidad alta, lo que representa el 75% del total y dos estudios presentaron calidad moderada, lo que representa el 25% del total. Los valores de OR (odd ratio) ^19^ para los factores evaluados muestran el; uso de magnificación regular ^20^ OR = 0,305, años de experiencia clínica ^20^ OR = 0,191; ausencia de calificación de posgrado OR = 3,04, cumplimiento deficiente con el uso de protección ocular OR = 2,52 y promedio de horas de trabajo por día de más de 8 horas OR = 2,16. ^21^ (Tabla 4).

**Tabla 4:** Criterios de Lista de verificación de estudios cualitativos CASP y escala OR

Título	Factores	OR	CASP grade	Clasificación de calidad
Occupational injuries to dental personnel		--	4,5	Moderado
The incidence and prevention of ocular injuries in orthodontic practice		--	8,5	Alto
Occupational ocular accidents amongst Greek endodontists: A national questionnaire survey	- Uso de magnificación regular - Años de experiencia clínica	0,305 0,191	9,5	Alto
Knowledge and Awareness of Eye Flu among the Dentists and Dental Auxiliaries of Udaipur City, Rajasthan		--	9,5	Alto
Work-related ocular events among Nigerian dental surgeons		--	9,5	Alto
Occupational ocular incidents in dentists: a multicenter study in southwestern Saudi Arabia	- Ausencia de calificación de posgrado - Cumplimiento deficiente con el uso de protección ocular - Promedio de horas de trabajo por dia de más de 8 horas	3,04 2,52 2,16	9,5	Alto
Prevalence of ocular injuries, conjunctivitis, and musculoskeletal disorder-related issues as occupational hazards among dental practitioners in the city of Salem: A randomized cross-sectional study		--	7,5	Moderado
Ocular injuries among oral and maxillofacial surgeons: Have high risk or not? An overview of a two-centered experience		--	9,5	Alto

## DISCUSIÓN

En la atención de servicios de salud bucal, tanto el personal odontológico como el propio paciente están expuestos a diferentes problemas ocupacionales que pueden afectar su integridad. Por ello, el personal odontológico debe identificar los factores o riesgos para su prevención y protección ^22^. No obstante, los trastornos músculo- esqueléticos (TME), las lesiones cutáneas, las lesiones oculares y el estrés, son algunos de los problemas más prevalentes ^23^. Por ello, los problemas ocupacionales se han incrementado, a pesar de que en la actualidad utilizamos equipos digitales e innovadores que permiten una menor exposición y menos horas en la atención dental ^24^^-^^26^.

Aun cuando varios estudios muestran la prevalencia de los diferentes riesgos ocupacionales, los estudios de tipos lesiones oculares son pocos y, en su mayoría, provienen de Europa, Asia y África. Como describe el artículo de Porter ^27^, las lesiones permanentes no se generan por las medidas inmediatas de primeros auxilios y el uso de irrigantes oculares como solución salina estéril o agua; medicamentos como colirio o ungüento antibiótico; preparación tópica de corticosteroides, entre otros ^(28, 29)^.

En principio, resultó que el uso ocasional de protección ocular fue un factor asociado principal para generar lesiones. Bell y Clement ^30^^)^ argumentan que, al comparar lentes protectores normales en lentes con protección lateral, la protección de estos últimos sería mayor; por lo tanto, recomiendan su uso, pues ofrecen una protección casi completa en procedimientos dentales ^(11, 26)^. Incluso, deben poseer características como diseño ergonómico, visibilidad clara sin distorsiones y fácil desinfección ^29^. Se ha encontrado en el estudio de Sims ^29^ mayor conciencia por parte de los ortodoncistas respecto de las lesiones oculares, pues el 64,3% proveía protección ocular a sus pacientes habitualmente, mientras que el 27,8% no lo hacía. 

Aydil *et al*^31^ describen que las lesiones se producen, aproximadamente, la quinta parte de las veces que no se emplea protección ocular; no obstante, se previene el 97,67% de lesiones cuando se usa protección. Por su parte, Zarra ^32^ no encontró asociación entre la incidencia de lesiones oculares y el adecuado uso de protección ocular, probablemente porque el 82% de los encuestados empleaba una protección ocular adecuada.

Según Zarra ^32^, los factores sexo, edad, horas trabajadas por año y años de práctica clínica influyen en los accidentes oculares. Incluso los endodoncistas jóvenes con menor tiempo de práctica clínica desde su graduación podrían haber experimentado algún accidente en un periodo no menor de 5 años.

Por otra parte, existe una asociación entre la incidencia de un accidente ocular y las horas de trabajo prolongado; esto podría deberse a la mayor destreza y experiencia de los endodoncistas en un año. Del mismo modo, Revankar *et al*. ^33^ determinaron que los cirujanos dentales con horarios de trabajo de 6-8 h/día fueron el 54,35%, mientras que el 22,46% realizó su trabajo durante 1-5 h/día, el 23,06% trabajó durante 9-12 h/día y solo el 1,05% trabajó 12 h/día. Igualmente, Azodo ^34^ informa que la incidencia de lesiones oculares se asoció con la edad, el uso de lentes de protección y los años de práctica.

No obstante, el estudio realizado por Aydil *et al*^31^ expone que los factores edad, sexo o experiencia no son factores de riesgo de lesiones oculares. También, Al-Dharrab ^35^ manifiesta que los jóvenes tienen mayor cuidado en comparación con los mayores al usar equipos protección en las practicas dentales ^36^. Sin duda, encontramos que la edad, el sexo y la experiencia influyen en el número de lesiones oculares. Entendemos que, en los diferentes centros de educación odontológicos, se enseña un cumplimento de estrategias de manejo de EPP ^37^ y medidas de bioseguridad a los alumnos. 

Asimismo, existen otros factores que se relacionan con los ya mencionados, como ausencia de calificación de posgrado. Aydil *et al*. ^31^ determinaron que algunos cirujanos orales y maxilofaciales no tienen educación sobre los riesgos laborales, lo que puede generar mayor riesgo de lesiones. Alsabaani ^21^ revela que el riesgo de exposición ocular a la sangre y los fluidos del paciente se incrementa por la falta de titulación de posgrado y el incumplimiento adecuado de protección ocular. No obstante, encontramos una menor exposición de los riesgos oculares entre odontólogos con título de posgrado, debido a que cumplen las normas de prácticas laborales seguras, como resultado de su larga trayectoria en la atención odontológica. Incluso, se menciona el desarrollo de programas de educación para reducir las lesiones oculares, es decir, promover protocolos, capacitaciones y documentación permanente sobre los procedimientos de manera más ordenada y preventiva ^31^.

Se ha encontrado que la posición de práctica, es decir, estar sentado o parado, es un factor asociado con lesiones oculares. La elección del cambio de posición por parte del profesional es para generar su comodidad y realizar su trabajo más eficiente, a fin de alcanzar una distancia adecuada entre el campo operatorio y su visión. Sin embargo, esta postura de trabajo en la mayoría de los casos no es ergonómica ^38^.

Por ello, Lietz ^39^ recomienda el uso de sillón dental ergonómico con aumento, en comparación con sillones convencionales sin aumento, debido a que los primeros podrían soportar mejor la región lumbar y mantener la curvatura de la espalda baja. Revankar ^33^, en su estudio, señala que el 94,68% de los odontólogos prefiere trabajar sentado y el 24,56%, la posición de pie.

Otros factores asociados son la exposición a riesgos laborales, biológicos y fuerzas mecánicas. Según Revankar ^33^ los riesgos para la salud ocupacional se dividen en biológicos (contacto con bioaerosoles infecciosos, sangre, saliva), físicos (quemaduras, lesiones, problemas auditivos, escaldaduras) y químicos (toxicidad, hipersensibilidad, alergias a materiales). En un estudio reciente, Azodo ^34^ menciona que los cirujanos dentales se encuentran fatigados por diferentes problemas ocupacionales de origen físico, biológico, químico e inclusive psicológico, lo que sitúa la práctica odontológica como un empleo estresante y peligroso. Como resultado halló que el 72,3% de los encuestados señala que ha experimentado una lesión ocular ^34^.

En el presente estudio, los tipos de lesiones oculares comúnmente reportados en la práctica odontológica son la entrada de líquidos, como clorhexidina, alcohol, solución anestésica local, enjuague bucal de benzalconio, solución salina, suero, agua, liquido modelador, detergente; la entrada de cuerpos extraños o partículas, como fragmentos dentales, restauraciones, adhesivo, polvo de acrílico, cemento de banda, alambre de ligaduras, amalgamas, NaOCl, EDTA, material provisional, vidrio, fragmentos de instrumentos, cálculos, óxido de zinc, eugenol, pasta de pulir, metal, arenilla de conducto roto, contacto con guantes; las lesiones químicas, producidas por solución de grabado o agente esterilizador; la salpicadura de fluidos biológicos, como saliva, sangre o ambas, y líquido quístico; y la conjuntivitis y las laceraciones.

Según Porter ^27^, las lesiones oculares ocurrieron por la entrada de líquidos en el ojo (39,4%) y por sólidos o cuerpos extraños (39,4%). Sims ^29^ describe que las lesiones de tipo cuerpo extraño fueron las más prevalentes. Además, Zarra ^32^ menciona que el 73% de endodoncistas que experimentaron lesiones oculares fueron más comúnmente por cuerpos extraños como amalgama y NaOCl; pero un número reducido de endodoncias que participaron en las encuestas mencionaron que las salpicaduras oculares con fluidos biológicos.

Por otro lado, Bhat *et al*. ^40^ encuestaron a 80 dentistas y 72 auxiliares dentales; de estos, 67 (44,08%) informaron que habían tenido una experiencia de gripe ocular causante de conjuntivitis. Azodo ^34^ describe que, de 148 cirujanos dentales, se presentaron incidentes oculares por cuerpo extraño en el 37,8%; por salpicaduras, en el 12,2%; por ambos, en el 22,3%; por salpicadura de fluidos biológicos, como saliva, en el 27,1%, mezcla de saliva y sangre, en el 17,8%, y solo sangre, en el 8,4%.

Del mismo modo, Alsabaani ^21^*et al*. examinaron a 233 odontólogos, de lo cual obtuvieron que los incidentes oculares por cuerpo extraño fueron el 29,6% (58,0% por material de unión y 36,2% por partículas dentarias) y por salpicaduras de líquidos fueron el 51,1% (48,7% por saliva y 47,9% por mezcla de sangre y saliva). 

Aydil *et al*. ^31^, en su investigación, hallaron que hubo contacto con salpicaduras de fluidos, tales como sangre (20,50%), saliva (24,84%), suero (24,22%) y una mezcla (30,43%). Además, hubo una contaminación por cuerpo extraño, que fue el 57,76%. Sin embargo, no mencionan el tipo laceración ocular. De modo similar, las lesiones causadas por contactos líquidos y cuerpos extraños fueron de mayor frecuencia en comparación con las lesiones múltiples no registradas. Finalmente, en la investigación de Revankar ^33^, los problemas relacionados con lesiones oculares sumaron el 52,6%; la conjuntivitis, el 27%; la abrasión corneal, el 5%; y la lesión química, el 4,3%.

La producción de evidencias con respecto al tema de las lesiones oculares que se originan en la práctica odontológica es escasa, debido a que gran parte de los estudios proviene de Reino Unido, Grecia, India, Nigeria, Arabia Saudita y Turquía. Por tanto, recomendamos continuar con este tema de investigación en Latinoamérica, para tener información más relevante y real.

## CONCLUSIONES

La mayoría de los estudios revisados demuestran que las lesiones oculares son atribuidas a factores asociados comunes y a los diferentes tipos de lesiones que ocurren en la práctica odontológica. Además, este estudio muestra que hubo una deficiencia en el cumplimiento de protocolos y educación sobre protección ocular y facial. 

## Fuente de financiamiento:

Autofinanciado

## References

[1] Ayers KM, Thomson WM, Newton JT, Morgaine KC, Rich AM (2009). Self-reported occupational health of general dental practitioners. Occup Med (Lond).

[2] Bârlean L, Danila I, Saveanu I, Balcos C (2013). Occupational health problems among dentists in Moldavian Region of Romania. Rev Med Chir Soc Med Nat Iasi.

[3] Díaz J, Chirinos M, Uribe J, Hilario J, Adrianzén R (2019). Características epidemiológicas de los traumatismos oculares en un instituto oftalmológico de referencia regional, Trujillo Perú, 2016-2017. Acta Méd Peru.

[4] Farrier SL, Farrier JN, Gilmour ASM (2006). Eye safety in operative dentistry - A study in general dental practice. British Dental Journal.

[5] World Health Organization (2020). Transmisión del SARS-CoV-2: repercusiones sobre las precauciones en materia de prevención de infecciones.

[6] Dockery DM, Rowe SG, Murphy MA, Krzystolik MG (2020). The Ocular Manifestations and Transmission of COVID-19: Recommendations for Prevention. J Emerg Med.

[7] Choi JH, Li Y, Kim SH (2018). The influences of smartphone use on the status of the tear film and ocular surface. PLoS One.

[8] Gowrisankaran S, Sheedy JE (2015). Computer vision syndrome: A review. Work.

[9] Nowrouzi-Kia B, Nadesar N, Sun Y, Gohar B, Casole J, Nowrouzi-Kia B (2020). Types of ocular injury and their antecedent factors: A systematic review and meta- analysis. Am J Ind Med.

[10] Ekmekcioglu H, Unur M (2017). Eye-related trauma and infection in dentistry. J Istanb Univ Fac Dent.

[11] American Academy of Ophthalmology (2020). COVID-19 y el cuidado de sus ojos.

[12] Labrie D, Moe J, Price RB, Young ME, Felix CM (2011). Evaluation of ocular hazards from 4 types of curing lights. J Can Dent Assoc.

[13] Alasiri R, Algarni H, Alasiri R (2019). Ocular hazards of curing light units used in dental practice - A systematic review. Saudi Dental Journal.

[14] Price RB, Labrie D, Bruzell EM, Sliney DH, Strassler HE (2016). The dental curing light: A potential health risk. J Occup Environ Hyg.

[15] Healthcare BV Critical Appraisal Skills Programme (CASP).

[16] Noyes J, Booth A, Flemming K (2018). Cochrane Qualitative and Implementation Methods Group guidance series-paper 3: methods for assessing methodological limitations, data extraction and synthesis, and confidence in synthesized qualitative findings. J Clin Epidemiol.

[17] Ricoy-Cano AJ, Obrero-Gaitán E, Caravaca-Sánchez F, Fuente-Robles YM Factors Conditioning Sexual Behavior in Older Adults: A Systematic Review of Qualitative Studies. J Clin Med.

[18] Herzog R, Álvarez-Pasquin MJ, Díaz C, Del Barrio JL, Estrada JM, Gil Á (2013). Are healthcare workers' intentions to vaccinate related to their knowledge, beliefs and attitudes? A systematic review. BMC Public Health.

[19] Aedo M S, Pavlov D S, Clavero Ch F (2010). Riesgo relativo y Odds ratio ¿Qué son y cómo se interpretan? Rev Obstet Ginecol Hosp. Santiago Oriente Dr. Luis Tisné Brousse.

[20] Zarra T, Lambrianidis T (2013). Occupational ocular accidents amongst Greek endodontists: a national questionnaire survey. Int Endod J.

[21] Alsabaani NA, Awadalla NJ, Abu Saq IH (2017). Occupational ocular incidents in dentists: a multicentre study in southwestern Saudi Arabia. Int Dent J.

[22] Moodley R, Naidoo S, Wyk JV (2018). The prevalence of occupational health-related problems in dentistry: A review of the literature. J Occup Health.

[23] Leggat PA, Kedjarune U, Smith DR (2007). Occupational health problems in modern dentistry: a review. Ind Health.

[24] Osazuwa-Peters N, Azodo CC, Obuekwe ON (2012). Occupational health issues of oral health care workers in Edo State, Nigeria. Int Dent J.

[25] Chochlidakis KM, Papaspyridakos P, Geminiani A, Chen CJ, Feng IJ, Ercoli C (2016). Digital versus conventional impressions for fixed prosthodontics: A systematic review and meta-analysis. J Prosthet Dent.

[26] Leggat PA, Kedjarune U, Smith DR (2007). Occupational health problems in modern dentistry: a review. Ind Health.

[27] Porter K, Scully C, Theyer Y, Porter S (1990). Occupational injuries to dental personnel. J Dent.

[28] Cronau H, Kankanala RR, Mauger T (2010). Diagnosis and management of red eye in primary care. Am Fam Physician.

[29] Sims AP, Roberts-Harry TJ, Roberts-Harry DP (1993). The incidence and prevention of ocular injuries in orthodontic practice. Br J Orthod.

[30] Bell KM, Clement DA (1991). Eye protection for the surgeon. J R Coll Surg Edinb.

[31] Aydil BA, Benlidayi ME, Kocaelli H, Dogancali GE, Genc A (2021). Ocular injuries among oral and maxillofacial surgeons: Have high risk or not? An overview of a two-centered experience. J Stomatol Oral Maxillofac Surg.

[32] Zarra T, Lambrianidis T (2013). Occupational ocular accidents amongst Greek endodontists: a national questionnaire survey. Int Endod J.

[33] Revankar VD, Chakravarthy Y, Naveen S, Aarthi G, Mallikarjunan DY, Noon AM (2019). Prevalence of Ocular Injuries, Conjunctivitis and Musculoskeletal Disorders-Related Issues as Occupational Hazards Among Dental Practitioners in the City of Salem: A Randomized Cross-Sectional Study. J Pharm Bioallied Sci.

[34] Azodo CC, Ezeja EB (2015). Work-related ocular events among Nigerian dental surgeons. Ann Occup Environ Med.

[35] (2012). Al-Dharrab AA, Al-Samadani KH. Assessment of hepatitis B vaccination and compliance with infection control among dentists in Saudi Arabia. Saudi Med J.

[36] (2014). Tada A, Watanabe M, Senpuku H. Factors influencing compliance with infection control practice in Japanese dentists. Int J Occup Environ Med.

[37] Villani FA, Aiuto R, Paglia L, Re D (2020). COVID-19 and Dentistry: Prevention in Dental Practice, a Literature Review. https://pubmed.ncbi.nlm.nih.gov/32604906/.

[38] Plessas A, Bernardes Delgado M (2018). The role of ergonomic saddle seats and magnification loupes in the prevention of musculoskeletal disorders. A systematic review. Int J Dent Hyg.

[39] Lietz J, Ulusoy N, Nienhaus A (2020). Prevention of Musculoskeletal Diseases and Pain among Dental Professionals through Ergonomic Interventions: A Systematic Literature Review. Int J Environ Res Public Health.

[40] Bhat N, Patel R, Reddy JJ, Singh S, Sharma A, Multani S (2014). Knowledge and Awareness of Eye Flu among the Dentists and Dental Auxiliaries of Udaipur City, Rajasthan. Int J Prev Med.

[41] Visscher KL, Hutnik CM, Thomas M (2009). Evidence-based treatment of acute infective conjunctivitis: Breaking the cycle of antibiotic prescribing. Can Fam Physician.

[42] Methley AM, Campbell S, Chew-Graham C, McNally R, Cheraghi-Sohi S (2014). PICO, PICOS and SPIDER: a comparison study of specificity and sensitivity in three search tools for qualitative systematic reviews. BMC Health Serv Res.

[43] Liberati A (2009). The PRISMA statement for reporting systematic reviews and meta-analyses of studies that evaluate health care interventions: explanation and elaboration. In Journal of clinical epidemiology.

[44] López A, Galparsoro DU, Fernández P (2001). Medidas de concordancia: el índice de Kappa. Cad Aten Primaria.

